# Notch2 Increases the Resistance to Venetoclax-Induced Apoptosis in Chronic Lymphocytic Leukemia B Cells by Inducing Mcl-1

**DOI:** 10.3389/fonc.2021.777587

**Published:** 2022-01-06

**Authors:** Stefania Fiorcari, Rossana Maffei, Claudio Giacinto Atene, Nicolò Mesini, Monica Maccaferri, Giovanna Leonardi, Silvia Martinelli, Ambra Paolini, Vincenzo Nasillo, Giulia Debbia, Leonardo Potenza, Mario Luppi, Roberto Marasca

**Affiliations:** ^1^ Department of Medical and Surgical Sciences, Section of Hematology, University of Modena and Reggio Emilia, Modena, Italy; ^2^ Hematology Unit, Department of Oncology and Hematology, Azienda-Ospedaliero Universitaria (AOU) of Modena, Policlinico, Modena, Italy; ^3^ Diagnostic Hematology and Clinical Genomics Laboratory, Department of Laboratory Medicine and Pathology, Azienda Unità Sanitaria Locale di Modena (AUSL)/Azienda Ospedaliero-Universitaria di Modena (AOU) Policlinico, Policlinico, Modena, Italy

**Keywords:** chronic lymphocytic leukemia (CLL), drug resistance, translational research, Notch, venetoclax (ABT-199)

## Abstract

Chronic lymphocytic leukemia (CLL) has experienced a clinical revolution—thanks to the discovery of crucial pathogenic mechanisms. CLL is still an incurable disease due to intrinsic or acquired resistance of the leukemic clone. Venetoclax is a Bcl-2 inhibitor with a marked activity in CLL, but emerging patterns of resistance are being described. We hypothesize that intrinsic features of CLL cells may contribute to drive mechanisms of resistance to venetoclax. We analyzed the expression of Interferon Regulatory Factor 4 (IRF4), Notch2, and Mcl-1 in a cohort of CLL patients. We evaluated CLL cell viability after genetic and pharmaceutical modulation of Notch2 expression in patients harboring trisomy 12. We tested venetoclax in trisomy 12 CLL cells either silenced or not for Notch2 expression or in combination with an inhibitor of Mcl-1, AMG-176. Trisomy 12 CLL cells were characterized by low expression of IRF4 associated with high levels of Notch2 and Mcl-1. Notch2 and Mcl-1 expression determined protection of CLL cells from spontaneous and drug-induced apoptosis. Considering the involvement of Mcl-1 in venetoclax resistance, our data demonstrated a contribution of high levels of Notch2 and Mcl-1 in a reduced response to venetoclax in CLL cells carrying trisomy 12. Furthermore, reduction of Mcl-1 expression by silencing Notch2 or by treatment with AMG-176 was able to restore the response of CLL cells to venetoclax. The expression of Notch2 identifies a subset of CLL patients, mainly harboring trisomy 12, characterized by high levels of Mcl-1. This biological mechanism may compromise an effective response to venetoclax.

## Introduction

Chronic lymphocytic leukemia (CLL) represents the most common leukemia in western countries. Clinically, CLL is characterized by a wide clinical heterogeneity ranging from patients with a highly stable disease for many years to patients with an aggressive disease (1). Although the deep reason of such clinical heterogeneity is not fully elucidated, it is mainly determined by genetic factors and by the complex relationship that leukemic cells entertain with the surrounding microenvironment. Among cytogenetic abnormalities, trisomy 12 (+12) arises in about 15%–18% of CLL cases. Patients who harbor +12 are characterized by more rapid disease progression, huge lymph node involvement, and increased prevalence of Notch1 mutations (2). In addition, acquisition of trisomy 12 occurs in one-third of CLL patients who develop Richter syndrome (3). In the past years, although progresses in understanding CLL pathogenesis have been achieved, the molecular events underlying the complex biology of trisomy 12 CLL are still under investigation.

Tyrosine kinase inhibitors and Bcl-2 inhibitors have deeply modified the treatment paradigm of CLL in either frontline or relapsed/refractory patients. In particular, venetoclax (ABT-199) is an oral, highly selective Bcl-2 inhibitor with demonstrated clinical efficacy across a different range of hematological malignancies. Venetoclax is a BH3-mimetic drug that selectively disrupts the interaction of Bcl-2 with BH3 domain proteins, triggering apoptosis (4). Among patients with untreated CLL, treatment with venetoclax was associated with longer progression-free survival (PFS) than that with standard treatment (5). In CLL patients with relapsed or refractory disease, venetoclax monotherapy shows an overall response of 80%, with a complete remission rate of 20% including 5% with undetectable minimal residual disease (MRD) (6, 7). Inferior PFS in refractory/relapsed CLL treated with venetoclax has been associated with Tumor Protein 53 (TP53) dysfunction, bulky adenopathy, Notch1 mutations, prior B-Cell Receptor (BCR) therapy failure, and complex karyotype (8, 9).

It has been demonstrated that clinical resistance may occur by intrinsic biological features of tumor cells or by acquired resistance that manifests after a patient has achieved a disease response and experiences disease progression while on therapy (10). Firstly, an acquired resistance mechanism to venetoclax has been identified as a mutation in Bcl-2 (Gly101Val), determining a reduction of drug binding to the hydrophobic groove. This mutation was not detected at treatment initiation but was found at disease progression (11). Secondly, the presence of significant levels of alternative pro-survival Bcl-2 family proteins results in resistance of CLL cells to venetoclax killing. In particular, high expression of Bcl-XL and Mcl-1 in CLL cells has emerged as an important contributor to reduced response (12). Considering all these pieces of evidence, identification of targetable vulnerabilities in the context of therapeutic resistance is a compelling need in CLL treatment.

Recently, we have demonstrated that CLL cells harboring +12 are characterized by a peculiar lower expression of IRF4 compared to other cytogenetic groups (13). IRF4 is a transcriptional regulator that exerts critical functions in different cell types of the immune cells (14). Deregulation of the biological programs controlled by IRF4 has been related to the pathogenesis of CLL (15–17). In particular, IRF4 is a critical regulator of Notch signaling during CLL development with elevated Notch2 expression that is linked to absence of IRF4 (18).

We demonstrated that patients harboring +12 are characterized by a higher amount of Notch2. The biological consequence of high expression of Notch2 is a stable maintenance of the antiapoptotic protein Mcl-1. Treatment of +12 CLL cells with venetoclax is impaired by elevated levels of Notch2 and consequently of Mcl-1. Here we report a new potential mechanism involved in reduced response to venetoclax treatment determined by maintenance of Mcl-1 expression through Notch2.

## Materials and Methods

### Chronic Lymphocytic Leukemia Patients and Samples

Blood samples were collected at diagnosis from CLL patients fulfilling standard clinical, morphological, and immunophenotypic criteria at the Division of Hematology of Modena with a protocol approved by the local institutional review board. Patient characteristics are listed in **Supplementary Table S1**. Peripheral blood mononuclear cells (PBMCs) collected from untreated CLL patients were isolated by density gradient centrifugation (Ficoll, Pharmacia LKB Biotechnology, Piscataway, NY, USA) and cryopreserved in RPMI-1640 medium, 50% fetal bovine serum (FBS), and 10% dimethyl sulfoxide (DMSO) and stored in liquid nitrogen until use. To enrich CLL cells, PBMCs were incubated with CD19 Microbeads (Miltenyi Biotech, Auburn, CA, USA), obtaining a purity of >99% as assessed by flow cytometry using Phycoerythrin (PE)-conjugated CD19 Ab (Miltenyi Biotech). All experiments were performed on highly purified CLL samples. CLL cells were treated with venetoclax (0.1 or 1 nM) or gliotoxin (100 nM) or AMG-176 (100 or 300 nM) or vehicle DMSO before evaluation (19–21).

### Real-Time PCR

RNA was extracted with the RNeasy Mini kit (Qiagen, Valencia, CA, USA). Then, RNA (100 ng) was reverse transcribed using SS Vilo Master Mix (Life Technologies). Ten nanograms of cDNA per reaction were analyzed in Real-Time PCR on Light Cycler 480 v.2 (Roche) in duplicate using SYBR Green Master Mix (Applied Biosystems). A housekeeping control [Glyceraldehyde-3-phosphate dehydrogenase (GAPDH)] was also amplified, and samples were analyzed by relative quantification methods.

### Transfection of Chronic Lymphocytic Leukemia B Cells

CD19+ CLL cells were transfected using either a plasmid vector or a small interfering RNA (siRNA). The Solution V kit was purchased from Lonza. The transfection was carried out in a Nucleofector (Lonza) with the P3 primary cell solution box using the program EO-117. Briefly, 5 × 10^6^ CD19+ CLL cells were transfected with 5 µg of IRF4 plasmid vector (RC504876, OriGene Technologies, Rockville/Maryland, USA) or 5 µg of the corresponding empty vector pCMV6-entry (PS100001, OriGene Technologies). The expression of IRF4 was silenced using a GeneSolution siRNA with 50 nM of each siRNA. A GeneSolution siRNA for Notch2 was used at a concentration of 50 nM. Non-targeted negative control siRNA (Qiagen) was used as a negative control. Transfected cells were subsequently plated in RPMI + 10% FBS and analyzed after 4, 24, and 48 h.

### Viability Assays

CLL cell viability was inspected in four experimental settings. In the first setting, viability of CD19+ CLL cells isolated from patients +12 was compared to viability of CLL cells isolated from patients without +12. CLL cells were cultured in RPMI + 10% FBS, and viability was tested at 24 and 48 h. In a second setting, +12 CLL cells were silenced using Notch2 siRNA or a non-targeted negative control siRNA, and viability was tested at 24 and 48 h. In a third setting, CLL cells were treated with venetoclax, and in some settings, with a combination of venetoclax and Notch2 siRNA. Lastly, CLL cells were treated with AMG-176 100 or 300 nM either in combination or not with venetoclax. Apoptotic cell death was analyzed using Annexin V-Fluorescein isothiocynate (FITC) and propidium iodide (PI) staining (eBioscience, San Diego, CA, USA). Events were acquired using a BD Accuri cytometer (Becton Dickinson) and then analyzed by FlowJo Software (RRID : SCR_008520).

### Flow Cytometry

CD19+ cells isolated from CLL patients were silenced for 24 h using non-targeted negative control siRNA and an IRF4 siRNA. CLL cells were collected and incubated with PE-conjugated anti-CD23 (Becton Dickinson, San José, CA, USA). For each sample, an isotype control was prepared in parallel.

### Immunoblotting

Purified CLL cells were transfected either with an empty vector and with an IRF4 plasmid vector for 24 h or silenced using non-targeted negative control siRNA and an IRF4 siRNA or Notch2 siRNA. In some settings, CLL cells were treated with venetoclax or gliotoxin. After 24 h, CLL cells were then lysed on ice for 10 min with lysis buffer supplemented with dithiothreitol and protease inhibitor cocktail (BioVision, Milpitas, CA, USA). Proteins (80 µg/lane) were electrophoresed on 4%–20% sodium dodecyl sulfate (SDS)-polyacrylamide gradient gels (Bio-Rad Laboratories, Hercules, CA, USA). Membranes were immunoblotted with primary antibodies listed in **Supplementary Table S2**. Then, membranes were incubated with species-specific horseradish peroxidase (HRP)-conjugated secondary antibody (diluted 1:50,000; GE Healthcare, Uppsala, Sweden) for 1 h and developed using HRP conjugates WesternBright Sirius (Advasta, Menlo Park, CA, USA). Images were acquired and analyzed using Image Lab Software v.3.0 (Bio-Rad Laboratories). Normalization analysis was performed by quantification of signals from the protein of interest and the housekeeping protein (actin) for all samples.

### Statistical Analyses

Data were analyzed using GraphPad Prism 6 (RRID : SCR_002798). In some experiments, results were normalized on control (100%) (vehicle-treated samples). Normalization was performed by dividing the value of a particular treated sample to the value of the corresponding sample treated with vehicle. p values were calculated by Student’s t-test or Mann–Whitney test (*p < 0.05, **p < 0.01). Data are presented as mean and standard error of the mean (SEM) is depicted as error bars.

## Results

### Increased Notch2 Level Characterized Trisomy 12 Chronic Lymphocytic Leukemia Cells

Recently, we have reported that reduced IRF4 expression distinguishes trisomy 12 CLL from other CLL cases, implying a possible involvement of IRF4 in the peculiar properties of this subset (13). We analyzed the level of IRF4 protein, confirming a lower expression of IRF4 in +12 CLL cells compared to no +12 (patient characteristics are listed in **Supplementary Table S1**). In our cohort of CLL cases harboring trisomy 12, 26% of patients displayed mutations in Notch1 genes (5/19 patients). We determined the differential expression of IRF4 in Notch1 unmutated and mutated samples and showed no significant difference between the two groups (**Supplementary Figure S1**, p = ns).

**Figure 1:**
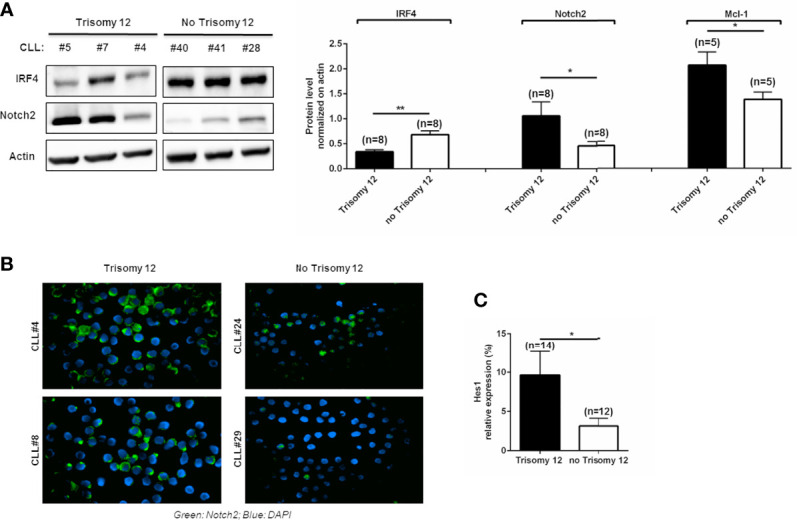
Trisomy 12 chronic lymphocytic leukemia (CLL) cells show low levels of IRF4 with higher expression of Notch2 and Mcl-1. **(A)** IRF4, Notch2, and Mcl-1 expression was measured by Western blot in CLL patients harboring trisomy 12 and with no trisomy 12. Blots represent three representative CLL patients harboring +12 and three without +12. On the right, bar diagrams represent the densitometric quantification of CLL samples with trisomy 12 and without trisomy 12 (*p < 0.05, **p < 0.01). **(B)** Immunostaining shows the expression of Notch2 in green and 4',6-diamidino-2-phenylindole (DAPI) in blue in two +12 and two no +12 CLL samples. **(C)** Hes1 expression was quantified in trisomy 12 vs. no trisomy 12 by real-time PCR (*p < 0.05).

IRF4 has been shown to be a critical regulator of Notch signaling in CLL cells, and Notch2 protein is the predominant Notch paralog expressed in IRF4^-/-^ CLL cells (18). Elevated amount of Notch2 protein in IRF4^-/-^ B cells was linked to changes in the posttranscriptional regulation (18, 22). We focused our attention on determining the expression of Notch2 protein in +12 and no +12 CLL cells, and we observed that +12 CLL cells with high expression of IRF4 are characterized by a significant increased expression of Notch2 compared to no +12 (**Figure 1A**; n = 8, *p < 0.05, **p < 0.01). The increased expression of Notch2 in CLL patients harboring trisomy 12 was also confirmed by immunofluorescence staining, as shown in **Figure 1B**. Notch signaling in CLL cells has a pro-survival activity that is explicated by the regulation of different targets as Mcl-1. Mcl-1 has an oncogenic role in CLL related to apoptosis resistance, poor prognosis, and chemoresistance (23, 24). We then investigated the expression of Mcl-1, showing that +12 CLL cells have higher levels of Mcl-1 compared to no +12 (**Figure 1A**; n = 5, *p < 0.05). On the contrary, we did not find any significant difference in Bcl-2 expression (data not shown). We also analyzed the transcriptional levels of another canonical Notch target gene, Hes1, known to be upregulated in CLL cells, finding the upregulation of Hes1 in trisomy 12 CLL cells (**Figure 1C**; *p < 0.05).

### IRF4 Modulates Notch2 and Mcl-1 Expression in Trisomy 12 Chronic Lymphocytic Leukemia Cells

**Figure 2:**
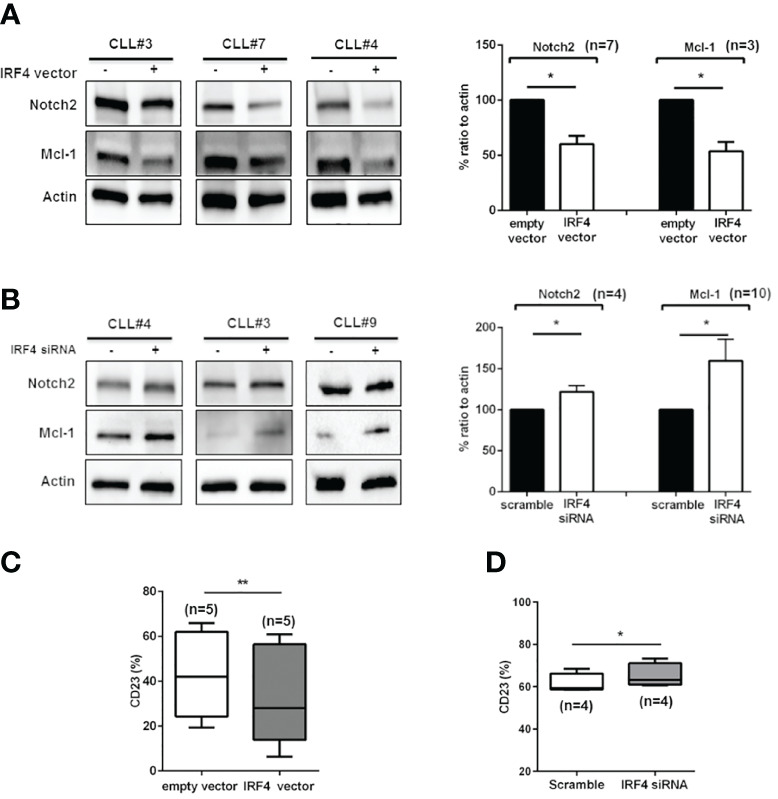
Notch2 and Mcl-1 expression in trisomy 12 is modulated by IRF4. **(A)** Purified trisomy 12 chronic lymphocytic leukemia (CLL) cells were transfected with an empty vector or with IRF4 vector and cultured in complete medium for 24 h. Immunoblots show Notch2 and Mcl-1 expression in three representative trisomy 12 CLL patients. On the right, bar diagrams show the densitometric quantification of Notch2 and Mcl-1 in +12 CLL cells transfected with an empty vector or IRF4 vector for 24 h (*p < 0.05). **(B)** Trisomy 12 CLL cells were silenced for IRF4 expression for 24 h. Immunoblots show the expression of IRF4 Notch2 and Mcl-1 after transfection with scramble or IRF4 siRNA in three representative samples. On the right, bar diagrams show densitometric quantification of Notch2 and Mcl-1 in +12 CLL cells after 24 h of transfection with IRF4 siRNA for 24 h (*p < 0.05, **p < 0.01). **(C)** Box plot shows the expression of CD23 measured by flow cytometry after transfection with empty or IRF4 vector for 24 h (**p < 0.01). **(D)** CD23 expression was measured after transfection of IRF4 siRNA or scramble control for 24 h (*p < 0.05).

To better characterize the influence of IRF4 expression in determining Notch2 expression in trisomy 12 CLL cells, transfections with IRF4 vector or siRNA were performed. As shown in **Figure 2**, forced upregulation of IRF4 determined a significant reduction of Notch2 levels together with an impairment of Mcl-1 expression (**Figure 2A**; *p < 0.05). On the contrary, silencing of IRF4 expression increased Notch2 and Mcl-1 upregulation (**Figure 2B**; *p < 0.05). Notch2 deregulation has been linked to aberrant expression of CD23 in CLL (25). On this line, CLL cells transfected with IRF4 vector showed a reduction of CD23 amount (**Figure 2C**; n = 5, *p < 0.05). On the contrary, CLL cells silenced for IRF4 expression displayed an increased expression of CD23 (**Figure 2D**; n = 4, *p < 0.05). These results clearly suggest that the low expression of IRF4 in +12 CLL cells is involved in the maintenance of Notch2 and its target genes.

### Notch2 Promotes the Survival of Trisomy 12 Chronic Lymphocytic Leukemia Cells

**Figure 3:**
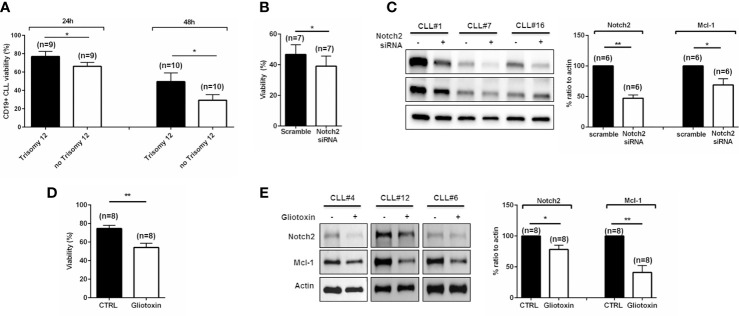
Notch2 is involved in trisomy 12 chronic lymphocytic leukemia (CLL) cell survival. **(A)** Bar diagrams show the percentage of CD19+ CLL cell viability cultured in complete medium for 24 and 48 h. Comparison was performed between trisomy 12 CLL cells and no trisomy 12 (*p < 0.05). **(B)** CLL cells were transfected with scramble or Notch2 siRNA, and bar diagram shows the viability measured after 24 h by flow cytometry (*p < 0.05). **(C)** Immunoblots show Notch2 and Mcl-1 levels after transfection with Notch2 siRNA in three representative samples. On the right, bar diagrams show densitometric quantification of Notch2 and Mcl-1 in trisomy 12 CLL cells after transfection with scramble or Notch2 siRNA (*p < 0.05). **(D)** CLL cells were cultured in the presence of gliotoxin, a specific inhibitor of Notch2. Bar diagrams show the percentage of CLL cell viability after 3 h of treatment (**p < 0.01). **(E)** Immunoblots show Notch2 and Mcl-1 levels after treatment with gliotoxin in three representative samples. Bar diagrams show the densitometric quantification of Notch2 and Mcl-1 in +12 CLL cells treated with gliotoxin (*p < 0.05, **p < 0.01).

We aimed to elucidate whether trisomy 12 and no trisomy 12 CLL cells are characterized by differential resistance to spontaneous apoptosis. We cultured both +12 and no +12 purified CLL cells in complete medium, and viability rate was assessed at 24 and 48 h. As shown in **Figure 3A**, +12 CLL cells were more resistant *in vitro* to cell death compared to no +12 (*p < 0.05). Given the important role of Notch2 signaling pathway to promote CLL cell survival, we wondered to determine its role in CLL cell maintenance. Notch2 expression was silenced by siRNA transfection, and CLL viability was assessed. Our data show that the reduction of Notch2 expression is able to induce an impairment of CLL cell viability (**Figure 3B**; n = 7, *p < 0.05). Of note, we observed a significant reduction of Mcl-1 expression due to Notch2 silencing (**Figure 3C**; n = 6, *p < 0.05, **p < 0.01). Gliotoxin is a fungal secondary metabolite that acts as a potent Notch2 transactivation inhibitor inducing apoptosis in CLL cells (21). We treated CLL cells with 100 nM of gliotoxin, and viability was assessed after 3 and 24 h of treatment. We monitored after 3 h a significant reduction of CLL cell viability that got worse after 24 h (**Figure 3D**; n = 8, *p < 0.05). Treatment with gliotoxin decreased the expression of Notch2 and significantly affected Mcl-1 already after 3 h, as shown in **Figure 3E**. These data confirmed the importance of Notch2 in preserving CLL cell survival.

### Trisomy 12 Chronic Lymphocytic Leukemia Cells Show Reduced Response to Venetoclax Treatment

**Figure 4:**
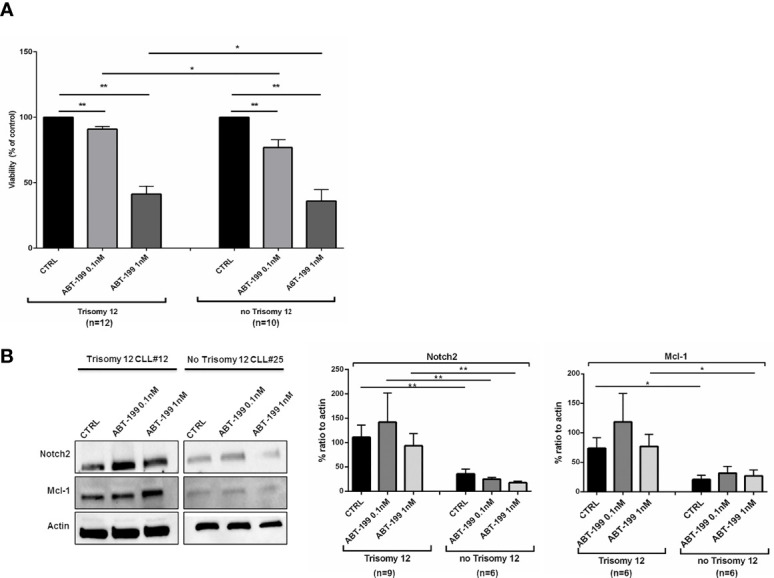
Trisomy 12 chronic lymphocytic leukemia (CLL) cells show a reduced response to venetoclax through Notch2/Mcl-1 axis. **(A)** Bar diagrams show comparison on viability rate of trisomy 12 vs. no trisomy 12 CLL cells in the presence of venetoclax at two different doses of 0.1 and 1 nM treatment for 24 h (*p < 0.05, **p < 0.01). **(B)** Immunoblots represent the level of Notch2 and Mcl-1 in one representative trisomy 12 CLL vs. no trisomy 12 CLL patient. On the right, bar diagrams show the densitometric quantification of Notch2 and Mcl-1 in +12 and no +12 CLL cells treated with venetoclax for 24 h (*p < 0.05).

Considering that Mcl-1 may contribute to decrease venetoclax-induced apoptosis (26), we aimed to determine the sensitivity of CLL cells to venetoclax treatment, comparing the response of CLL cells isolated from patients harboring trisomy 12 to no trisomy 12. CLL cells were treated in complete medium in either the presence or absence of venetoclax at doses of 0.1 or 1 nM. We measured an induction of apoptosis by venetoclax in +12 and in no +12 CLL cells. Comparison of viability rate between +12 and no +12 CLL samples displayed a differential induction to apoptosis, with no +12 CLL cells being more prone to undergo venetoclax apoptosis than +12 CLL cells (**Figure 4A**; n = 12 trisomy 12 and n = 10 no trisomy 12, *p < 0.05, **p < 0.01). To unravel the biological reason of this decreased response in +12 cells, we analyzed Notch2 and Mcl-1 expression during treatment with venetoclax. As shown in **Figure 4B**, +12 CLL cells were characterized by maintenance of stable levels of Notch2 and Mcl-1 during treatment with venetoclax (n = 9 for Notch2 and n = 6 for Mcl-1, *p < 0.05, **p < 0.01).

### Inhibition of Notch2 and Mcl-1 Increases Venetoclax Sensitivity of Trisomy 12 Chronic Lymphocytic Leukemia Cells

**Figure 5:**
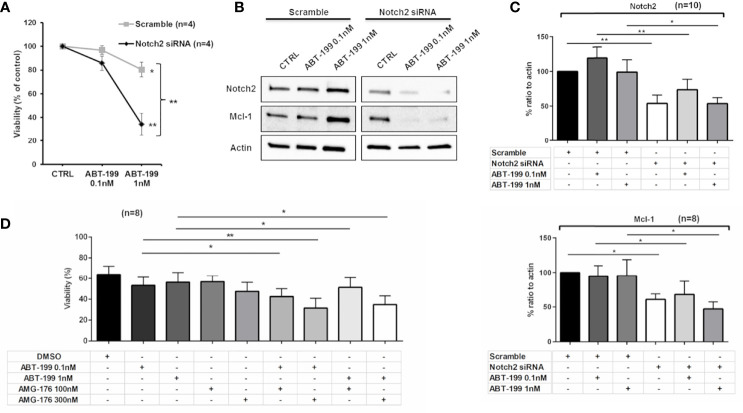
Combination of venetoclax and inhibition of Mcl-1 induces apoptosis in trisomy 12 chronic lymphocytic leukemia (CLL) cells. **(A)** Diagram shows the percentage of survival in CLL cells transfected with scramble or Notch2 siRNA in the presence of treatment with venetoclax for 24 h (*p < 0.05, **p < 0.01). **(B)** Immunoblots show the level of Notch2, Mcl-1 in one representative CLL patient harboring trisomy 12 during treatment with venetoclax after transfection with either scramble control or Notch2 siRNA (*p < 0.05, **p < 0.01). **(C)** Bar diagrams depict the densitometric quantification of Notch2 and Mcl-1 in CLL cells harboring trisomy 12 transfected with scramble or Notch2 siRNA either in combination or not with venetoclax (*p < 0.05, **p < 0.01). **(D)** Trisomy 12 CLL cells were either treated with dimethyl sulfoxide (DMSO) or treated with 0.1 or 1 nM venetoclax, 100 and 300 nM AMG-176, or a combination of both for 48 h. Bar diagrams show the viability (%) in each condition measured by flow cytometry (*p < 0.05, **p < 0.01).

In order to assess the contribution of Mcl-1 in determining a reduction to venetoclax response, we compared the viability of +12 CLL cells transfected with Notch2 siRNA or scramble control both treated with venetoclax for 24 h. We observed a significant reduction of CLL cell viability when venetoclax was combined with Notch2 siRNA compared to combination with scramble control (**Figure 5A**; n = 4, *p < 0.05, **p < 0.01).

Analysis of Notch2 signaling suggests that reduced levels of Mcl-1, induced by Notch2 silencing, may influence the sensibility of CLL cells to venetoclax treatment (**Figures 5B, C**; *p < 0.05, **p < 0.01). Altogether, our data display the involvement of Notch2/Mcl-1 axis in determining a reduced response to venetoclax in CLL cells. Since inhibition of Mcl-1 is effective in CLL cells and the Mcl-1 antagonist, AMG-176, has been demonstrated to induce apoptosis in CLL setting (20), we evaluated the combination of venetoclax with AMG-176 to impair trisomy 12 CLL cell viability. Leukemic cells isolated from +12 CLL patients were incubated with venetoclax (0.1 or 1 nM), 100 or 300 nM AMG-176, or combination of both. As shown in **Figure 5D**, after 48 h, we detected a significant induction of apoptosis by dual inhibition of Bcl-2 and Mcl-1 compared to treatment with venetoclax alone (n = 6, *p < 0.05, **p < 0.01).

## Discussion

Venetoclax (ABT-199) is the first Food and Drug Administration (FDA)-approved highly selective oral Bcl-2 antagonist for the treatment of CLL. It displaces proapoptotic BH3-only proteins from Bcl-2, facilitating activation of proapoptotic BAX or BAK proteins to induce apoptosis (27). In CLL, venetoclax shows high response rates, even in traditionally high-risk subgroups and is able to induce deep remissions with undetectable measurable residual disease (MRD) and prolonged PFS (28). Some open questions about venetoclax treatment in CLL are related to the prevention of resistant clones. Herein, we demonstrate a new mechanism that may determine reduced response to venetoclax in CLL patients harboring trisomy 12. This subgroup of CLL patients displays low levels of IRF4 and elevated expression of Notch2, implying the upregulation of its target genes as Mcl-1 and CD23. The maintenance of high levels of Notch2 in +12 CLL cells and consequently of Mcl-1 enforces the survival and the possibility of these cells to escape the proapoptotic effect of venetoclax.

Notch receptors play a critical role in the pathogenesis of different hematological malignancies, regulating different biological functions related to tumorigenesis, proliferation, differentiation, and apoptosis. Notch1, Notch2, and their ligands Jagged1 and Jagged2 are constitutively expressed and activated in CLL cells compared to normal B lymphocytes (23). Increased activity of Notch1 pathway is implicated in CLL maintenance and evolution, protecting leukemic cells from apoptosis, promoting proliferation, and inducing genes of the BCR and cytokine/chemokine signaling (29, 30). Different studies have reported frequent Notch1 gene alterations that lead to a truncated protein. Clinically, Notch1 mutation is a genetic marker that identifies a high-risk group of CLL patients characterized by Richter transformation and poor outcome (31). Moreover, Notch2 is also consistently expressed by CLL cells and is involved in survival maintenance through regulation of the survival molecule CD23 (23, 25). Furthermore, Notch2 supports leukemic cell viability through the maintenance of the antiapoptotic protein Mcl-1 expression (32). This evidence is confirmed by treating CLL cells with gliotoxin, a fungal secondary metabolite, that is a potent Notch2 inhibitor. Gliotoxin induces apoptosis in CLL leukemic cells, interfering with Notch2 signaling (21). Among the complexities of CLL, different studies have highlighted the role of IRF4 in its development. In particular, Shukla et al. (18) established IRF4 as a critical regulator of Notch2 signaling during CLL development, supporting a role for Notch2 in leukemia initiation. This study has identified deregulation of IRF4–Notch axis as a major pathway in the molecular pathogenesis of CLL (18). IRF4-/- B cells are characterized by increased activation of Notch pathway with no detectable levels of Notch3 and Notch4 and no significant differences in Notch1. Therefore, Notch2 is strongly upregulated with no difference in transcriptional activation but at posttranscriptional levels (18, 22). Nedd4 is an ubiquitin ligase and represents a target gene of IRF4 that has been shown to degrade Notch receptors (18).

In this scenario and based on our previous study, we focused our attention on CLL patients harboring trisomy 12 who are characterized by low levels of IRF4 (13). Trisomy 12 is present in approximately 16% of cases of CLL, and the acquisition of this cytogenetic abnormality is related to pronounced lymphadenopathy and splenomegaly and higher rates of cell proliferation, disease progression, and Richter syndrome transformation (33, 34). Our data demonstrate a correlation between low expression of IRF4 and high levels of Notch2 in trisomy 12 CLL patients. Elevated expression of Notch2 masters the amount of Mcl-1 and promotes protection of +12 CLL cells to apoptosis that is not observed in the other patients. This is in line with the observation that Notch signaling may control Mcl-1, regulating its biosynthesis (32). The functional evidence of the involvement of Notch2 in preserving survival of trisomy 12 CLL cells is given by the genetic silencing or pharmaceutical inhibition of its expression by gliotoxin that demonstrates an impairment of Mcl-1 expression and consequently a reduction of leukemic cell viability. In light of the above results, we wondered to determine the sensitivity of CLL cells to treatment with venetoclax *in vitro*, discriminating between patients with and without trisomy 12. Our findings indicate that +12 CLL cells display a reduced response to venetoclax compared to the others, given the high levels of Notch2 and Mcl-1. These results are in line with previous observations that demonstrate that a primary resistance to this drug may be driven by kinase signaling cascades as CD40L and BCR stimulation that determine upregulation of BCL-X_L_, Mcl-1, and Bfl-1, suggesting a possible involvement of lymph node microenvironment in affecting sensitivity to selective Bcl-2 inhibition (19). To define the impact of Notch2/Mcl-1 axis in affecting the response to venetoclax, we silenced Notch2 in CLL cells, observing an increase in proapoptotic effect of venetoclax. Since it has been demonstrated that CLL cells show resistance to venetoclax given high levels of Mcl-1, we combined the Bcl-2 inhibition with an Mcl-1 antagonist. AMG-176 is a first-in-class Mcl-1 inhibitor in clinical development for hematologic malignancies that has shown activity in inducing CLL cell death and has shown an additive or synergistic effect in combination with venetoclax (20, 35). The possibility to target CLL clone by combining different therapeutic agents needs to be considered. As previously reported, BCR pathway inhibitors and anti-CD20 monoclonal antibodies may overcome venetoclax resistance by affecting Mcl-1 induction (19, 36).

Some crucial points need to be dissected and deepened. At this time, there is no significant evidence derived from clinical trials suggesting a clear worse outcome in patients with trisomy 12 treated with venetoclax. Moreover, no data are reported concerning the impact of trisomy 12 aberration on PFS or on the rate of complete response (CR) and MRD negativity during venetoclax treatment (8). Nevertheless, a more specific analysis should be performed, also considering that an association with a worse outcome was observed in patients treated at relapse with venetoclax and the monoclonal antibody (MoAb) rituximab in patients carrying Notch1 mutations that are present at high frequency in trisomy 12 CLL cases (37). A longer follow-up is necessary to better evaluate the clinical impact of +12 aberration in venetoclax treatment response.

Of relevance, tumor microenvironment may determine an additional support in maintaining the expression of Notch2 and Mcl-1, especially at the lymph node level. This is in line with emerging clinical data that suggest an involvement of lymph node niches to induce resistance to proapoptotic treatments (38).

Altogether, our data suggest the presence of a new intrinsic mechanism involved in suboptimal response to venetoclax resulting from low IRF4 expression that may contribute to the development of venetoclax resistance. Further studies are needed to confirm the involvement of the IRF4/Notch2 axis also in CLL population characterized by low IRF4 expression without +12 aberration. Moreover, further analysis on the potential use of IRF4/Notch2 expression as a predictive marker to venetoclax response should be explored as well as the combination of targeted strategies to circumvent and/or reverse this mechanism of resistance.

## Data Availability Statement

The original contributions presented in the study are included in the article/**Supplementary Material**, further inquiries can be directed to the corresponding authors.

## Ethics Statement

The studies involving human participants were reviewed and approved by Comitato Etico Provinciale; ce 175/2014, October 17, 2014. The patients/participants provided their written informed consent to participate in this study. The study was conducted in full conformance with the principles of the Declaration of Helsinki.

## Author Contributions

SF, RMaf, and RMar conceived the research. SF coordinated the research and interpreted the results. SF analyzed the results and wrote the article. SF and CA performed the *in vitro* experiments. SF acquired and analyzed flow cytometric data. SF and CA performed the statistical analyses. NM, SM, MM, AP, VN, GL, and GD provided sample evaluation. RMar revised critically and approved the final version of the article. LP and ML revised and approved the final version of the paper. All authors contributed to the article and approved the submitted version.

## Funding

This work was supported by Associazione Italiana per la Ricerca sul Cancro (IG14376 and IG21436 to RMar), Associazione Italiana per la Ricerca sul Cancro and Fondazione Cariplo (TRIDEO 16923 to RMaf). Progetto Eccellenza 2018-2022. SF was supported previously by Triennial AIRC/FIRC Fellowship 16430, Milan, Italy, and by Fondazione Umberto Veronesi, Milan, Italy. The funders had no role in study design, data collection and analysis, decision to publish, or preparation of the article.

## Conflict of Interest

RMar received research funding from Janssen and Gilead Sciences and honoraria from Gilead Sciences, Janssen, Abbvie, Roche, and Shire. ML received honoraria from Gilead Sciences, MSD, and Pfizer. RMaf has received speaker fee from Abbvie.

The remaining authors declare that the research was conducted in the absence of any commercial or financial relationships that could be construed as a potential conflict of interest.

## Publisher’s Note

All claims expressed in this article are solely those of the authors and do not necessarily represent those of their affiliated organizations, or those of the publisher, the editors and the reviewers. Any product that may be evaluated in this article, or claim that may be made by its manufacturer, is not guaranteed or endorsed by the publisher.
