# Supplementary material for: Notch2 Increases the Resistance to Venetoclax-Induced Apoptosis in Chronic Lymphocytic Leukemia B Cells by Inducing Mcl-1
Source: Front Oncol. 2022 Jan 6;11:777587. doi: 10.3389/fonc.2021.777587 (PMC8770925; doi:10.3389/fonc.2021.777587)
Supplement: Supplementary file 1 [file DataSheet_1.docx]

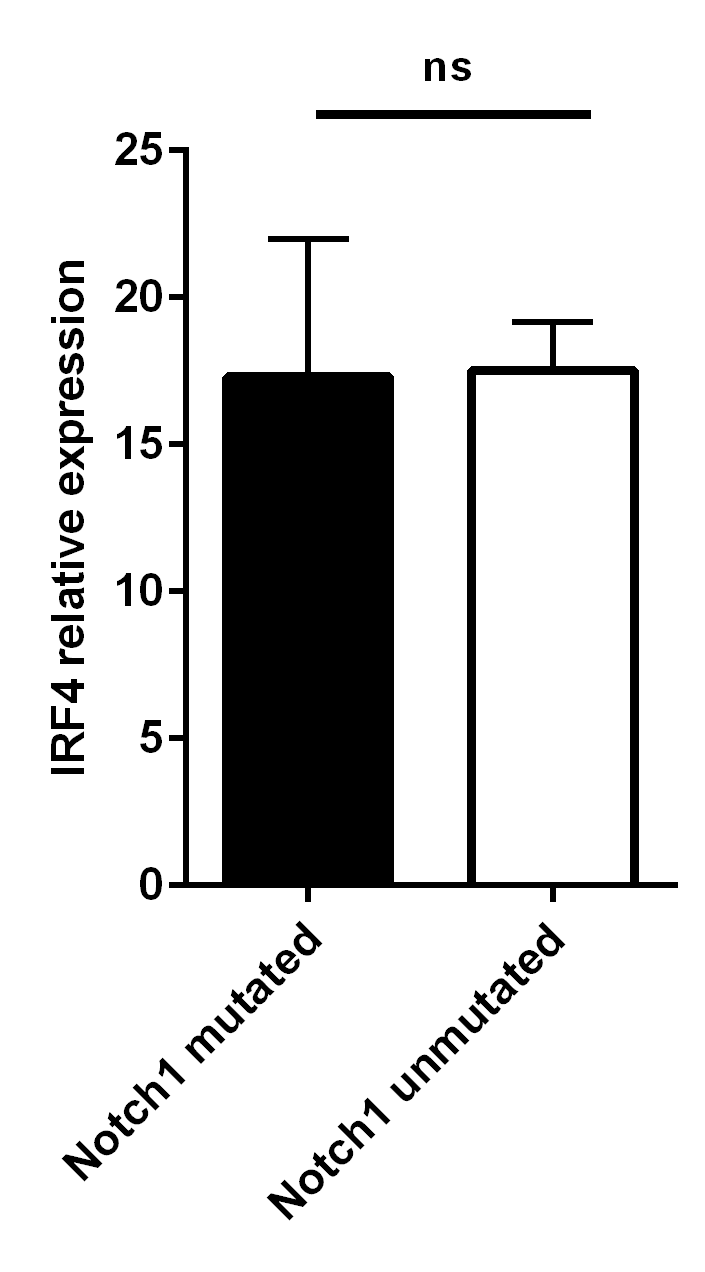


**Supplementary Figure 1.** IRF4 expression was quantified in Notch1 mutated versus unmutated Notch1 CLL cells by Real-time PCR (p=ns)
