# Supplementary material for: Notch2 Increases the Resistance to Venetoclax-Induced Apoptosis in Chronic Lymphocytic Leukemia B Cells by Inducing Mcl-1
Source: Front Oncol. 2022 Jan 6;11:777587. doi: 10.3389/fonc.2021.777587 (PMC8770925; doi:10.3389/fonc.2021.777587)
Supplement: Supplementary file 2 [file Table_1.docx]

**Supplementary Table S1.**

**Patients Characteristics**

| **CLL#** | **Gender** | **Age at diagnosis** | **Binet stage at diagnosis** | **IGHV mutational status** | **NOTCH1 mutational status** | **FISH** | **Previous treatment at sample collection** |
| --- | --- | --- | --- | --- | --- | --- | --- |
| **1** | F | 78 | A | mutated | unmutated | trisomy 12 | no |
| **2** | M | 80 | A | unmutated | unmutated | trisomy 12 | no |
| **3** | F | 49 | A | mutated | unmutated | trisomy 12 | no |
| **4** | M | 68 | A | unmutated | unmutated | trisomy 12 | no |
| **5** | M | 35 | C | na | unmutated | trisomy 12 | no |
| **6** | F | 57 | A | unmutated | mutated | trisomy 12 | no |
| **7** | M | 70 | A | unmutated | mutated | trisomy 12 | no |
| **8** | M | 79 | A | mutated | unmutated | trisomy 12 | no |
| **9** | F | 63 | A | unmutated | mutated | trisomy 12 | no |
| **10** | M | 72 | A | unmutated | mutated | trisomy 12 | no |
| **11** | M | 60 | B | unmutated | unmutated | trisomy 12 | no |
| **12** | M | 77 | A | mutated | unmutated | trisomy 12 | no |
| **13** | M | 66 | A | mutated | unmutated | trisomy 12 | no |
| **14** | M | 69 | A | mutated | unmutated | trisomy 12 | no |
| **15** | M | 57 | A | mutated | mutated | trisomy 12 | no |
| **16** | M | 39 | B | na | na | trisomy 12 | no |
| **17** | M | 89 | A | mutated | unmutated | trisomy 12 | no |
| **18** | M | 59 | A | unmutated | unmutated | trisomy 12 | no |
| **19** | F | 74 |  | mutated | unmutated | trisomy 12 | no |
| **20** | M | 69 | A | unmutated | na | del13q | no |
| **21** | M | 64 | A | mutated | unmutated | del13q | no |
| **22** | M | 43 | A | mutated | unmutated | normal | no |
| **23** | F | 68 | A | mutated | na | normal | no |
| **24** | M | 42 | B | unmutated | na | del17p | no |
| **25** | M | 52 | A | unmutated | unmutated | del13q | no |
| **26** | M | 71 | C | unmutated | unmutated | del13q | no |
| **27** | F | 47 | A | mutated | unmutated | normal | no |
| **28** | M | 54 | A | unmutated | na | normal | no |
| **29** | F | 68 | A | mutated | unmutated | na | no |
| **30** | F | 78 | A | mutated | unmutated | del11q | no |
| **31** | M | 58 | A | mutated | na | normal | no |
| **32** | F | 52 | A | mutated | unmutated | del13q | no |
| **33** | F | 57 | B | mutated | na | del11q | no |
| **34** | F | 53 | A | unmutated | na | normal | no |
| **35** | M | 53 | B | unmutated | na | del13q | no |
| **36** | M | 53 | B | unmutated | unmutated | normal | no |
| **37** | M | 61 | A | unmutated | na | normal | no |
| **38** | F | 74 | A | mutated | unmutated | del13q | no |
| **39** | M | 39 | C | unmutated | unmutated | del13q | no |
| **40** | M | 71 | A | na | unmutated | del11q | no |
| **41** | F | 59 | A | unmutated | na | del13q | no |
| **42** | M | 64 | A | mutated | na | normal | no |
