# Supplementary material for: Notch2 Increases the Resistance to Venetoclax-Induced Apoptosis in Chronic Lymphocytic Leukemia B Cells by Inducing Mcl-1
Source: Front Oncol. 2022 Jan 6;11:777587. doi: 10.3389/fonc.2021.777587 (PMC8770925; doi:10.3389/fonc.2021.777587)
Supplement: Supplementary file 3 [file Table_2.docx]

**Supplementary Table 2.**

**Antibodies used in immunoblotting**

| **Antibody** | **Manufacturer** |
| --- | --- |
| **Anti-IRF4 (#4964S)** | Cell Signaling Tech, Beverly, MA |
| **Anti-Notch2 (#5732)** | Cell Signaling Tech, Beverly, MA |
| **Anti-Mcl-1 (#4572)** | Cell Signaling tech, Beverly, MA |
| **Anti-βactin mouse (#6276)** | Abcam, Cambridge, UK |
